# Distinct Mechanisms of IgM Antibody-Mediated Acquired von Willebrand Syndrome and Successful Treatment with Recombinant von Willebrand Factor in One Patient

**DOI:** 10.1159/000522236

**Published:** 2022-01-27

**Authors:** Matthias Höpting, Ulrich Budde, Andreas Tiede, Matthias Grube, Joachim Hahn, Wolfgang Herr, Susanne Heimerl, Christina Hart

**Affiliations:** ^a^Department of Hematology and Oncology, Internal Medicine III, University Hospital Regensburg, Regensburg, Germany; ^b^Medilys Laborgesellschaft mbH, Asklepios Klinik Hamburg-Altona, Hamburg, Germany; ^c^Department of Hematology, Hemostasis, Oncology and Stem Cell Transplantation, Hannover Medical School, Hannover, Germany; ^d^Institute of Clinical Chemistry and Laboratory Medicine, University Hospital Regensburg, Regensburg, Germany

**Keywords:** Bleeding disorders, Paraproteinaemia, Acquired von Willebrand syndrome, von Willebrand factor, Monoclonal gammopathy of undetermined significance

## Abstract

Acquired von Willebrand Syndrome (AVWS) is a rare coagulation disorder which can be associated with IgM paraproteinaemia. Recently, recombinant von Willebrand factor (rVWF) has become available for the treatment of bleedings in patients with inherited von Willebrand disease, but experience in patients with AVWS is limited. We report on 2 patients with AVWS with underlying IgM paraproteinaemia with distinct underlying pathomechanisms. In 1 patient, the paraprotein built unspecific complexes with von Willebrand factor (VWF). In the other patient, we were able to detect an IgM antibody against VWF. Bleeding in this patient was successfully treated with rVWF. To our knowledge, this is the first report about the successful use of rVWF in a patient with AVWS with the detection of a VWF-specific antibody.

## Introduction

Acquired von Willebrand syndrome (AVWS) is a rare coagulation disorder often caused by cardiovascular or lymphoproliferative diseases and can be associated with severe mucosal bleedings. In patients with underlying IgM paraproteinaemia, three different pathological mechanisms are known to cause AVWS: (i) specific neutralizing antibodies that interfere with von Willebrand factor (VWF) function, (ii) specific non-neutralizing antibodies that increase VWF clearance, and (iii) unspecific antibodies that build complexes with VWF [1].

Causal treatment has been reported to resolve the bleeding disorder. Treatment options for acute bleeding include desmopressin, antifibrinolytics, and plasma-derived (pd)VWF [1]. Recently, recombinant von Willebrand factor (rVWF) has become available for the treatment of bleedings and prevention of surgical bleedings in patients with inherited von Willebrand disease; however, experience in patients with AVWS is limited [2].

Here, we report on 2 patients with AVWS and associated IgM paraproteinaemia with distinct pathological mechanisms causing VWF activity impairment. One patient was treated with rVWF, showing an improved incremental recovery of VWF activity and clinical response compared to prior treatment with pdVWF.

## Case Report

**Fig. 1:**
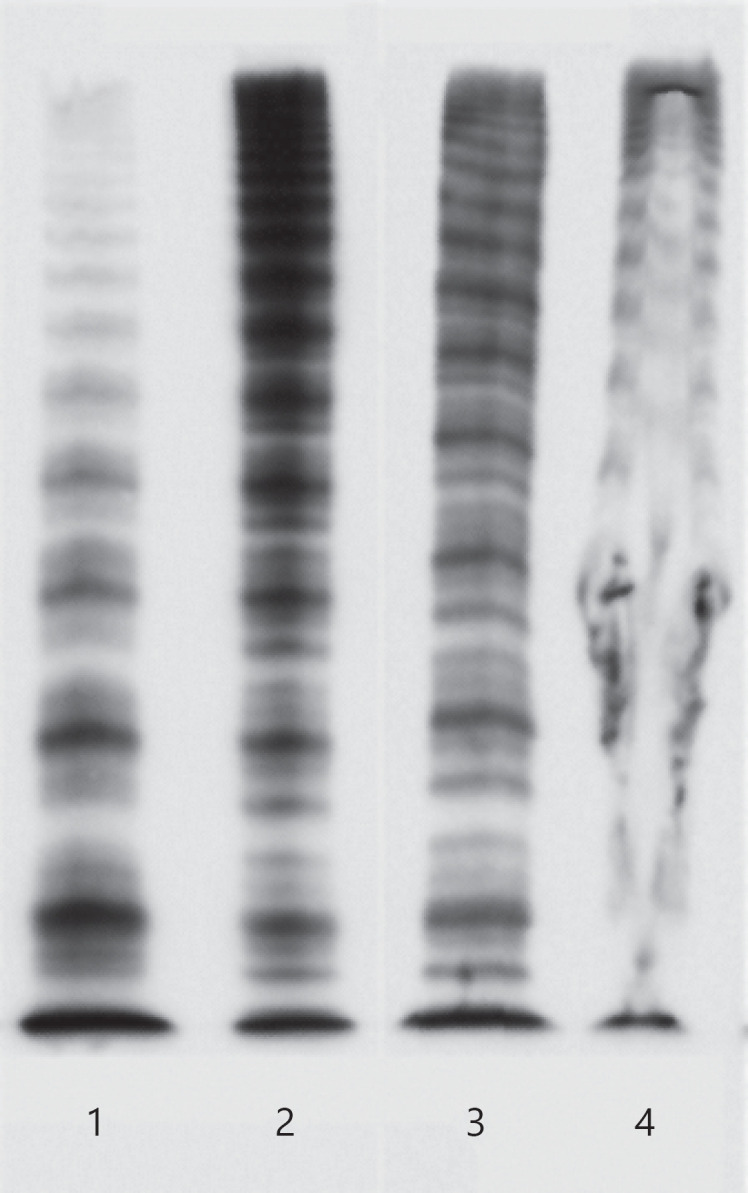
Multimer analysis of patient's plasma at presentation. Column 1 shows multimer analysis of patient 1 indicating the loss especially of the HMWMs. Columns 2 and 3 show normal patient's plasma. Column 4 shows multimer analysis of patient 2 with a disruption of the bands caused by complexes built with monoclonal IgM antibodies.

Patient 1, an 80-year-old man, presented with recurrent severe transurethral bleeding and a hemoglobin level of 6.4 g/dL. No previous bleeding in his own or family history was reported. The laboratory work-up at presentation showed reduced VWF antigen and VWF activity with a pathological ratio of VWF activity/antigen of 0.2 (lower limit of normal ≥0.7) (Table 1). Platelet light transmission aggregometry (LTA, Born method, APACT 4S Plus, Fa Greiner) showed no response upon stimulation with ristocetin. Immunofixation revealed the presence of an IgM kappa monoclonal gammopathy, while the total IgM level was normal (Table 1). Multimer analysis of VWF showed a loss of most of the high molecular weight multimers (HMWMs) and also of some intermediate molecular weight multimers (Fig. 1). ELISA analysis proved the existence of a VWF-specific IgM antibody, whereas mixing studies did not show any impairment of VWF activity. Due to the elevated bleeding risk, a bone marrow biopsy was not performed.

Therapy was initiated with simultaneous treatment with tranexamic acid (500 mg every 8 h intravenously), intravenous immunoglobulin (1g/kg body weight/day, 2 days), and substitution of pdVWF/factor VIII (FVIII) products. The first administration of 2500IU pdVWF/FVIII (40 IU/kg/day) led to an increase of VWF activity from 6% to 52% (after 1 h), but after 4 h VWF activity fell to 26% and after 24 h to 16%. Despite increasing doses of pdVWF up to 160 IU/kg/day, transurethral bleeding did not resolve.

We decided to treat the patient with rVWF (vonicog alfa, Takeda GmbH) which is especially rich in ultra-large VWF multimers and HMWMs [3]. We observed an increased recovery after 2 h after the first infusion of vonicog alfa (42 IU/kg) from 32% to 75%. 12 h after the second infusion of vonicog alfa (42 IU/kg), VWF activity was still 59%. Transurethral bleeding stopped and did not recur.

Immunosuppressive treatment was immediately started with prednisolone (1 mg/kg body weight/day) and rituximab (375 mg/m^2^ body surface area, weekly, 4 cycles), but VWF activity did not improve and bleeding still did not resolve. Even though bleeding had stopped finally after treatment with vonicog alfa, we decided to treat the patient with bortezomib (1.3 mg/m^2^ body surface area) on days 1, 4, 8, and 11 and dexamethasone 20 mg on days 1, 2, 4, 5, 8, 9, 11, 12 because of persisting decreased VWF activity. Bortezomib has been previously reported to induce long-term remission in patients with AVWS associated with IgG paraproteinaemia [4]. After only one cycle of treatment, VWF activity increased to 200% and immunofixation did not show any sign of monoclonal gammopathy anymore. Two more consolidating cycles of bortezomib/dexamethasone were administered, and the patient remained in long-term remission for 1 year after the initial bleeding.

Patient 2, a 71-year-old man, presented with epistaxis and severe intestinal bleeding after resection of a colonic polyp which required erythrocyte concentrate transfusion. In his own or family history, no previous bleeding was reported. The laboratory testing at presentation showed reduced VWF antigen and VWF activity with a normal ratio (VWF activity/antigen) of 1.28 (≥0.7). FVIII activity was somewhat reduced (Table 1). Platelet LTA showed a slightly reduced response with all agonists (adenosine diphosphate [ADP], collagen, ristocetin, arachidonic acid) (Table 1). Immunofixation revealed IgM lambda monoclonal gammopathy with an elevated total IgM level (Table 1). Multimer analysis of VWF showed a severely distorted band structure and a destroyed agarose gel (Fig. 1). Primarily, the patient did not agree to any therapy or further diagnostic work-up. Two months later, he fell on his hip and developed a large hematoma. At the time of hospitalization, laboratory diagnostic showed VWF antigen 17%, VWF activity 24%, and FVIII activity 38%. Total IgM was further increased to >6,000 mg/dL. Flow cytometry of the peripheral blood showed monoclonal B cells, and a monoclonal B-cell lymphocytosis was diagnosed. Treatment with bendamustine and rituximab was initiated. After 4 cycles of treatment, IgM and VWF activity reached normal values. The bleeding did not occur again.

## Discussion

In summary, we describe 2 cases of patients with AVWS with underlying IgM paraproteinaemia and two distinct mechanisms of VWF impairment. In patient 1, we found low concentrations of monoclonal IgM and a VWF-specific autoantibody, increasing the clearance of VWF. Multimer analysis showed a significant loss primarily of HMWMs (Fig. 1). Ristocetin was not able to induce platelet aggregation due to the loss of HMWMs. RVWF concentrate was more effective than pdVWF, possibly because of its higher content in HMWM VWF. HMWMs are the most important VWF multimers for primary hemostasis [5]. Patient 1 was refractory to causal treatment with prednisolone, immunoglobulin, and rituximab. Intravenous immunoglobulin administration had been described as a potential treatment option in IgM monoclonal gammopathy of undetermined significance (MGUS) [6] and rituximab in IgG-MGUS [7]. Later on, the patient showed immediate and sustainable response to dexamethasone and bortezomib as described earlier for patients with AVWS and IgG multiple myeloma [4].

In contrast, patient 2 was diagnosed with high concentrations of IgM paraprotein. Anti-VWF autoantibodies were not detected. Multimer analysis showed distorted structures throughout the range of multimer sizes suggesting the formation of unspecific complexes of VWF and the paraprotein (Fig. 1). Treatment with immunoglobulin had been described to be often unsuccessful in patients with this underlying pathomechanism [1]. Binding of IgM paraprotein seems to be unspecific [8]. Likely, immunoglobulin treatment is only seldom successfully used in these AVWS patients because the unspecific IgM paraprotein also rapidly binds infused immunoglobulins so that they do not have any protective effect on VWF concentration and activity. As previously described, treatment of the underlying disease resolved the AVWS associated with IgM paraproteinaemia [1].

Furthermore, we report on the use of vonicog alfa, a rVWF product, in a patient with evidence of a specific antibody against VWF. In this setting, experience with rVWF is very limited. So far, it has been successfully used in a patient with AVWS with an underlying cardiac disease [9]. It has been previously described that the administration of rVWF in patients with MGUS and smoldering myeloma was not effective [3].

In our case, the use of rVWF was promising because especially the HMWMs were lost and vonicog alpha is rich in ultra-large multimers and HMWMs. In contrast to pdVWF products, rVWF is not cleaved by ADAMTS13 during the production process [3]. Primarily, vonicog alfa had a good recovery rate after infusion in our patient. But VWF activity was decreasing gradually under substitution with rVWF similar to the use of plasmatic products. However, bleeding did not occur again. It might be speculated that even the transient presence of HMWMs and ultra-large VWF multimers might have prevented further bleeding, especially since they are cleaved rapidly by ADAMTS13 after secretion into the bloodstream in a physiological situation anyway. Further research is required to investigate this hypothesis.

In the literature, 1 patient with elevated FVIII levels and AVWS developed an arterial embolic stroke under substitution with a pdVWF/FVIII product [10]. In a systematic review, 2 patients were found that suffered from major venous thromboembolism associated with FVIII containing infusions. Both patients had elevated FVIII levels [11] pointing out that thromboembolism in patients with von Willebrand disease is a rare complication but might occur in the presence of elevated FVIII levels. RVWF does not contain FVIII and therefore might decrease the risk of thromboembolism after VWF substitution.

In conclusion, the application of rVWF in our patient with a VWF-specific IgM antibody was safe, resulted in a sufficient increase of VWF activity, and stopped bleeding. As previously reported, rVWF showed only a suboptimal response in a patient with an inhibitory antibody [3]. It remains unclear whether rVWF will work in patients with monoclonal IgM antibodies building complexes with VWF. In summary, rVWF can be considered as a treatment option for bleeding in patients with AVWS and specific antibodies against VWF. However, the use in this setting should be monitored carefully and experience should be reported.

## Statement of Ethics

Written informed consent was obtained from both patients for publication of their data in this case report and any accompanying images. According to the Ethics Committee of the University of Regensburg, ethics approval was not required.

## Conflict of Interest Statement

A.T. received grants or honoraria for lectures or consultancy from Biotest, CSL Behring, Octapharma, and Takeda. All other authors declare no conflicts of interest.

## Funding Sources

No author received funding related to the preparation of this article.

## Author Contributions

M.H. analyzed the data and wrote the manuscript. C.H. and S.H. analyzed the data and performed critical manuscript review and edits. U.B., M.G., and J.H. provided important data and performed critical manuscript reviews and edits. A.T. provided important intellectual input and performed critical manuscript review and edits. W.H. performed critical manuscript review and edits.

## Data Availability Statement

All data analyzed during this study are included in this article. Further inquiries can be directed to the corresponding author.

## Figures and Tables

**Table 1:** Patient characteristics and laboratory results at presentation

Parameter	Patient 1	Patient 2
Age	80	71
Gender	Male	Male
Underlying disease	Monoclonal IgM paraproteinaemia	Monoclonal B-cell lymphocytosis
Paraprotein	IgM kappa	IgM lambda
Serum concentration of paraprotein, mg/mL	94 [40–230]	513 [40–230]
Platelets, /nL	288 [163–337]	193 [163–337]
APTT, ^†^ sec	40 [26–37]	46 [26–37]
FVIII:C, ^‡^ %	29 [70–150]	30 [70–150]
VWF: Ag, ^§^%	27 [57–174]	17 [57–174]
VWF: Ac, ^¶^%	6 [47–173]	22 [47–173]
Platelet LTA (born), %	ADP^††^: 89 [>70]	ADP^††^: 43 [>70]
	Collagen: 82 [>70]	Collagen: 68 [>70]
	Ristocetin: no agglutination [>70]	Ristocetin: 54 [>70]
	Arachidonic acid: 85 [>70]	Arachidonic acid: 46 [>70]
VWF plasma multimers	Type 2	Severe type 1
Anti-VWF antibody	Positive	Negative
Proposed mechanism	Increased VWF clearance	Nonspecific complex formation

Normal values are shown in brackets.

† APTT, activated partial thromboplastin time.

‡ FVIII:C, factor VIII, clotting activity.

§ VWF: Ag, von Willebrand factor antigen.

¶ VWF: Ac, von Willebrand factor activity.

†† ADP, adenosine diphosphate.
